# Supplementary material for: Regulator of Lipid Metabolism NHR-49 Mediates Pathogen Avoidance through Precise Control of Neuronal Activity
Source: Cells. 2024 Jun 4;13(11):978. doi: 10.3390/cells13110978 (PMC11172349; doi:10.3390/cells13110978)
Supplement: Supplementary file 1 [file cells-13-00978-s001.zip › FigS2_legend.pdf]

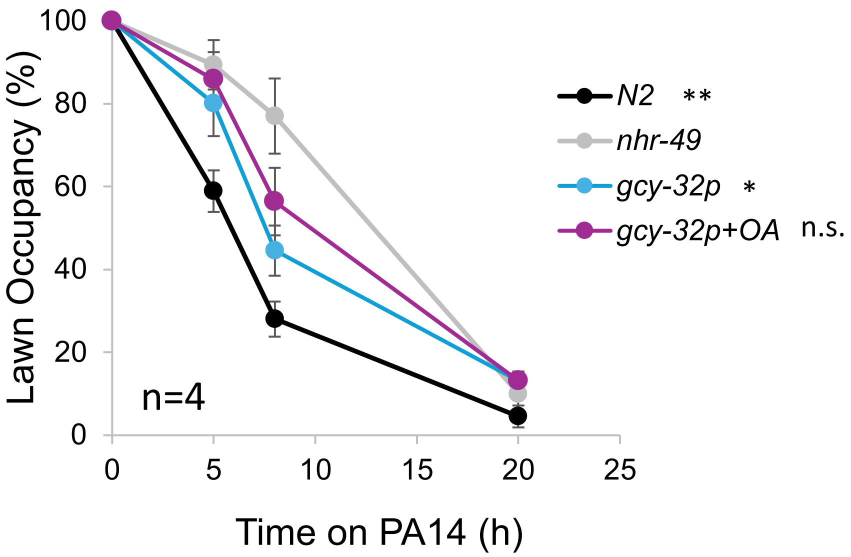

Figure S2. Lawn occupancy of body cavity neuron rescue strains of *nhr-49*. PA14 avoidance of body cavity neuron rescue transgenic strain supplemented with 300 μM OA. Asterisks indicate p value (*<0.05, **<0.01) as determined by one-way ANOVA with Tukey's multiple comparisons test.
