# Supplementary material for: Regulator of Lipid Metabolism NHR-49 Mediates Pathogen Avoidance through Precise Control of Neuronal Activity
Source: Cells. 2024 Jun 4;13(11):978. doi: 10.3390/cells13110978 (PMC11172349; doi:10.3390/cells13110978)
Supplement: Supplementary file 1 [file cells-13-00978-s001.zip › Figure S1_legends.pdf]

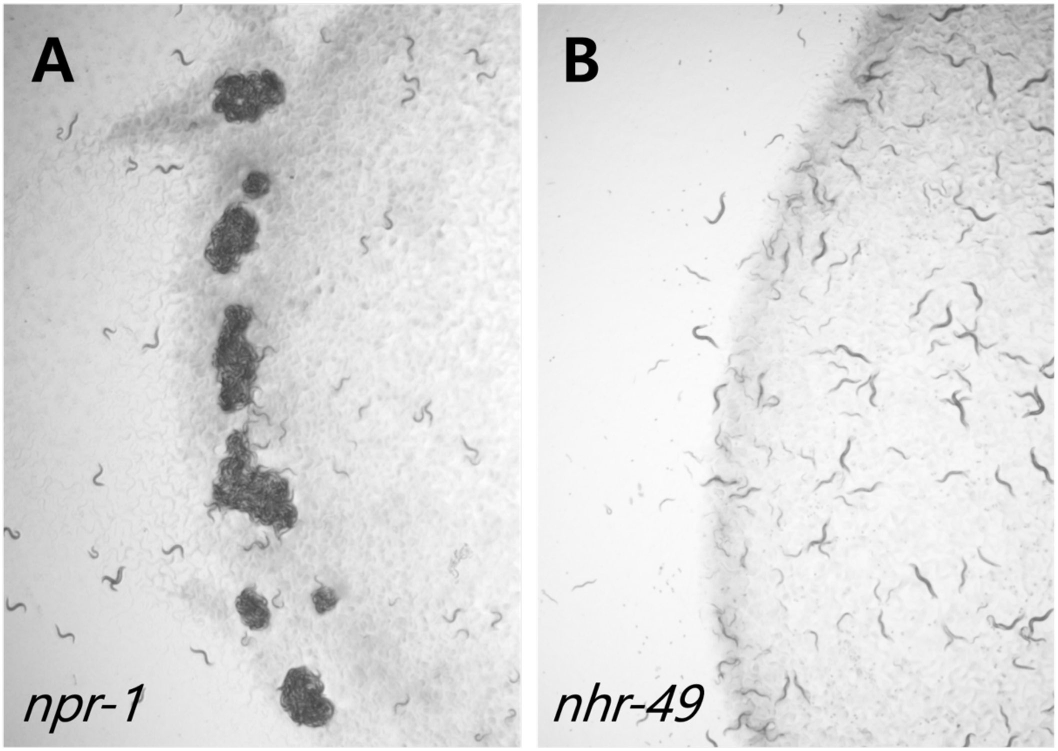

Figure S1. Comparison of oxygen related behaviors between *npr-1* and *nhr-49* mutants. (A) *npr-1* mutant worms can typically be seen collecting at the border of bacterial lawns (‘bordering’) in aggregated clumps. (B) Absence of such behaviors in *nhr-49* mutants.
