# Supplementary material for: Regulator of Lipid Metabolism NHR-49 Mediates Pathogen Avoidance through Precise Control of Neuronal Activity
Source: Cells. 2024 Jun 4;13(11):978. doi: 10.3390/cells13110978 (PMC11172349; doi:10.3390/cells13110978)
Supplement: Supplementary file 1 [file cells-13-00978-s001.zip › Table S1_S2_revision.pdf]

Table S1. All strains used for this study

Experimental Models: Organisms/strains	Source	Identifier
nhr-49(nr2041)	CGC	STE68
nhr-49(nr2041); okyEx104[rgef-1p::nhr-49::SL2::gfp]	(1)	KHY140
nhr-49(nr2041); wbmEx149[ges-1p::3xHA::nhr-49(cDNA)::unc-54 3'UTR + myo-3p::mCherry]	CGC	WBM409
nhr-49(nr2041); okyEx109[myo-3p::nhr-49 + myo-2p::mCherry]	(1)	KHY216
nhr-49(nr2041); okyEx110[unc-17p::nhr-49::SL2::gfp]	(1)	KHY161
nhr-49(nr2041); okyEx114[eat-4p::nhr-49::SL2::gfp]	(1)	KHY183
nhr-49(nr2041); okyEx115[tph-1p::nhr-49::SL2::gfp]	(1)	KHY179
nhr-49(nr2041); okyEx116[dat-1p::nhr-49::SL2::gfp]	(1)	KHY180
daf-3(e1376)	CGC	CB1376
daf-3(e1376);nhr-49(nr2041)	This study	KHY244
nhr-49(nr2041); okyEx101[gcy-32p::nhr-49::SL2::gfp + unc-122p::mCherry]	This study	KHY228
nhr-49(nr2041); okyEx120[gcy-32p::et7::SL2::gfp + unc-122p::mCherry]	This study	KHY206
nhr-49(nr2041); okyEx122[gcy-32p::et8::SL2::gfp + unc-122p::mCherry]	This study	KHY218
nhr-49(nr2041); okyEx123[gcy-32p::et13::SL2::gfp + unc-122p::mCherry]	This study	KHY228
nhr-49(nr2041); gcy-36(ok2208)	This study	KHY245
lin-15B&lin-15A(n765); qaIs2241[gcy-36p::egl-1 + gcy-35::gfp]	CGC	CX7102
nhr-49(nr2041); qaIs2241[gcy-36p::egl-1 + gcy-35::gfp]	This study	
nhr-49(nr2041); okyEx126[gcy-32p::tetx + unc-122p::gfp]	This study	KHY208
okyEx108[gcy-32p::GCaMP6s + unc-122p::mCherry]	This study	KHY242
nhr-49(nr2041); okyEx108[gcy-32p::GCaMP6s + unc-122p::mCherry]	This study	
nhr-49(nr2041); okyEx130[gcy-32p::nhr-49::SL2::mCherry]; oky108[gcy-32p::GcaMP6s + unc-122p::mCherry]	This study	KHY243

Table S2. All primers used for this study.

Cloning Primers	Forward 5'-3'	Reverse 5'-3'
Pgcy-32	aaGCATGCTGGGGAAGACGATAGACGGCG	TCTATAATACAATCGTGATCTTCGCTTC
tetx	attattGCTAGCATGCCGATCACCATCAACAACTT	aataatGGTACCTTAAGCGGTACGGTTGTACAGGT
GCaMP6	attattGGATCCATGGGTTCTCATCATCATCATCATCA	aatcatGAATTCTCACTTCGCTGTCATCATTTGTACA

Mutagenesis Primers	Forward 5'-3'	Reverse 5'-3'

et7	CTTGCAGCTCTATTGGCAATTC	AGTTGCTGAGAGCATTCC
et8	GCTCCGGTCTTTTTACAGCAAC	CGTCGAATGATCATTGATGTC
et13	TTGGCAGAGGAGGATTCTCTG	TCCGAAGAACTTTGTAAGTG

**qPCR primers**	**Forward 5'-3'**	**Reverse 5'-3'**
**act-1**	ACGACGAGTCCGGCCCATCC	GAAAGCTGGTGACGATGGTT
**daf-7**	CCAAGGGGTGCTGCTTGTAT	TTCCGCCAAGTTGAAGTGGT
**flp-18**	CGGAAACGTGATGTGCCAA	CGCTTGTCGAACGAATCACG
**flp-21**	CCGGCAGTGACAGAGTTTAC	TCCAAATCGGAGAGGACGAG

1. Kwon S, Park KS, Yoon KH. Dissecting the Neuronal Contributions of the Lipid Regulator NHR-49 Function in Lifespan and Behavior in C. elegans. Life (Basel). 2023;13(12).
