# Supplementary figures and images for: Regulator of Lipid Metabolism NHR-49 Mediates Pathogen Avoidance through Precise Control of Neuronal Activity
Source: Cells. 2024 Jun 4;13(11):978. doi: 10.3390/cells13110978 (PMC11172349; doi:10.3390/cells13110978)

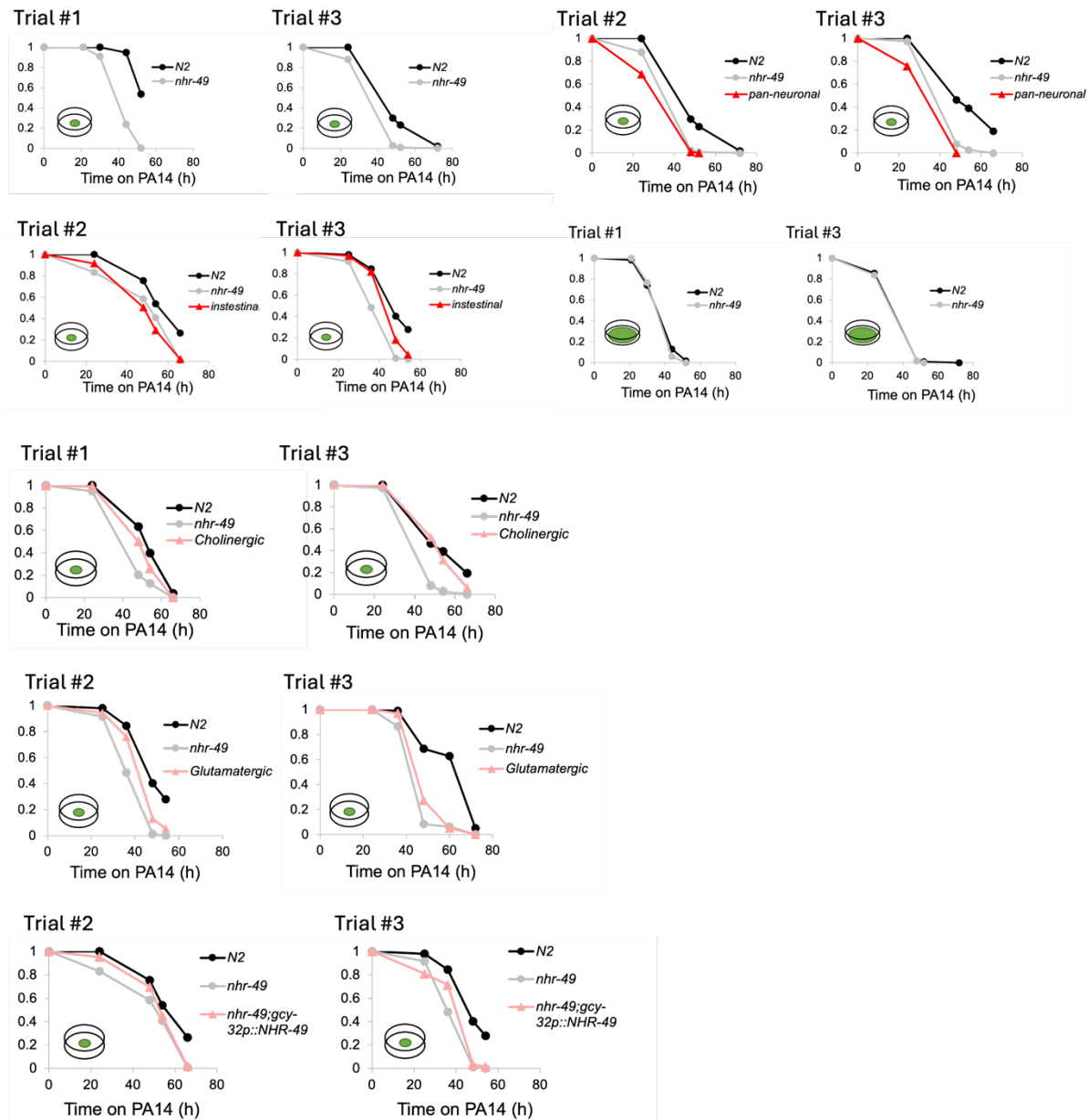

**Figure S3. Graphs of all trials of PA14 survival assays.** Raw data can be found in Table S3.

Supplement: Supplementary file 1 [file cells-13-00978-s001.zip › Figure S3_legend.pdf]
